# Supplementary material for: Atomic-scale mapping of hydrophobic layers on graphene and few-layer MoS2 and WSe2 in water
Source: Nat Commun. 2019 Jun 13;10:2606. doi: 10.1038/s41467-019-10740-w (PMC6565678; doi:10.1038/s41467-019-10740-w)
Supplement: Supplementary file 1 — Supplementary Information [file 41467_2019_10740_MOESM1_ESM.pdf]

Supplementary Information

Atomic-scale mapping of hydrophobic layers on graphene and few-layer MoS2 and WSe2 in water

Manuel Ralph Uhlig, Daniel Martin-Jimenez and Ricardo Garcia

Materials Science Factory, Instituto de Ciencia de Materiales de Madrid, CSIC,
c/ Sor Juana Ines de la Cruz 3, 28049 Madrid, Spain

E-mail: r.garcia@csic.es

Supplementary note 1

The parameters of the cantilevers used in the experiments are listed below.

Supplementary Table 1. Cantilever parameters (second eigenmode).

	k_2 [N m^{-1}]	Q_2	f_{r2} [kHz]	σ_2 [nm V^{-1}]
SiC/graphene	1126	20.4	961	11.53
MoS_2	1126	20.4	961	11.53
WSe_2	1051	26.5	957	13.25
$MoSe_2$	2338	19.4	918	7.17
WS_2	2338	19.4	918	7.17
Mica	1128	19.5	978	10.89
HOPG	969	22.7	904	14.05

Supplmentary note 2

Supplementary Figure 1 shows some lattice-resolution (phase shift) images of the 2D materials immersed in water. Two-dimensional fast Fourier transforms (2D FFT) have been applied to obtain the crystal structure of the surfaces. The obtained lattice parameters are indicated in the figures.

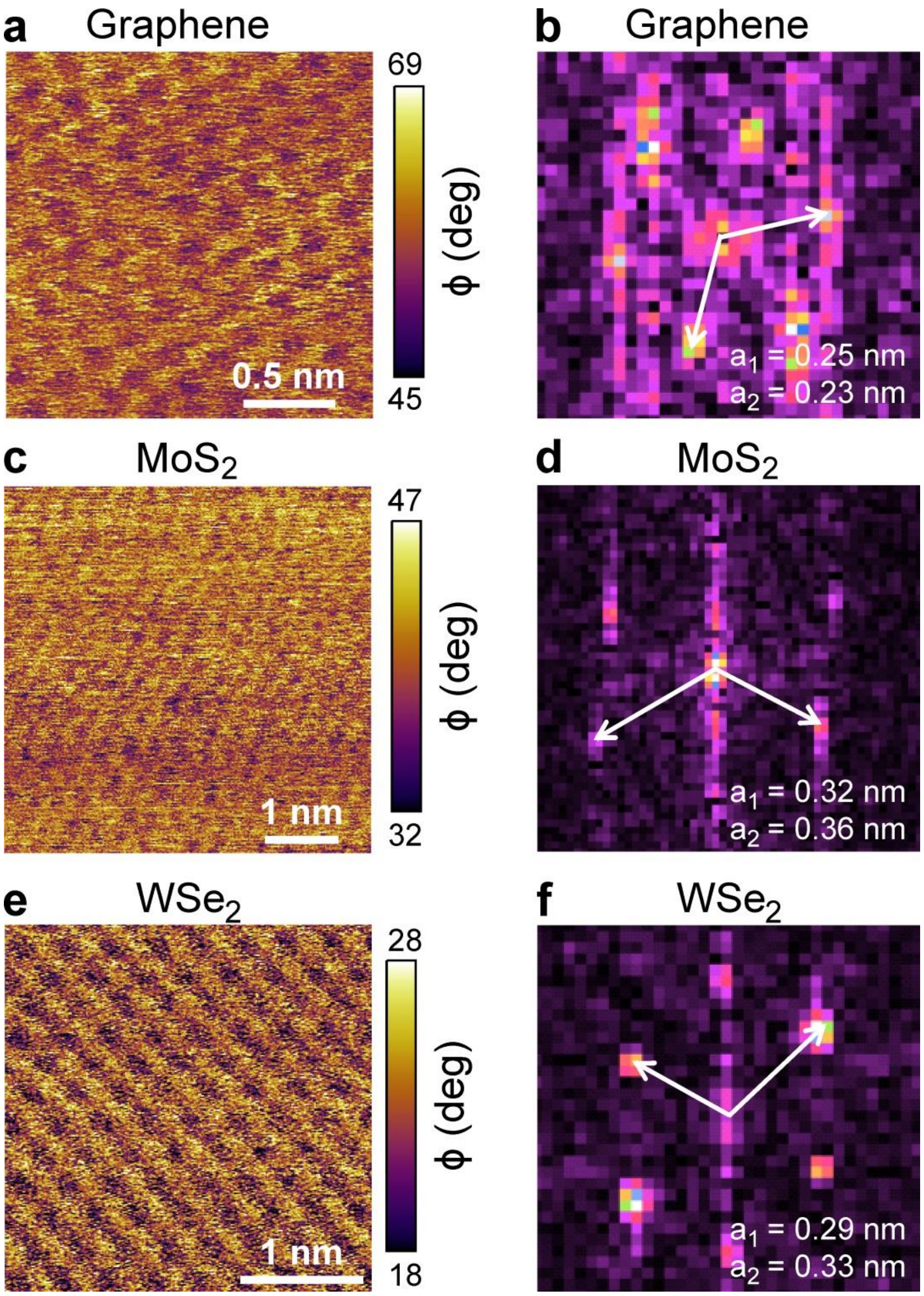

Supplementary Figure 1. Lattice resolution images of 2D materials in water**.** (a) AFM phase image of the lattice structure of graphene. (b) Fast Fourier transform (FFT) of (a). (c) AFM phase image of (few-layer) MoS_2. (d) FFT of (c). AFM phase image of WSe_2. (f) FFT of (e). The AM-AFM was operated with a free amplitude A_0= 240 pm, with an amplitude set-point A_{sp} between 0.30 and 0.50 A_0. The imaging was performed at a scan rate of 10 to 20 Hz.

Supplementary note 3

3D-AFM images of MoS_2 and WS_2-water interfaces.

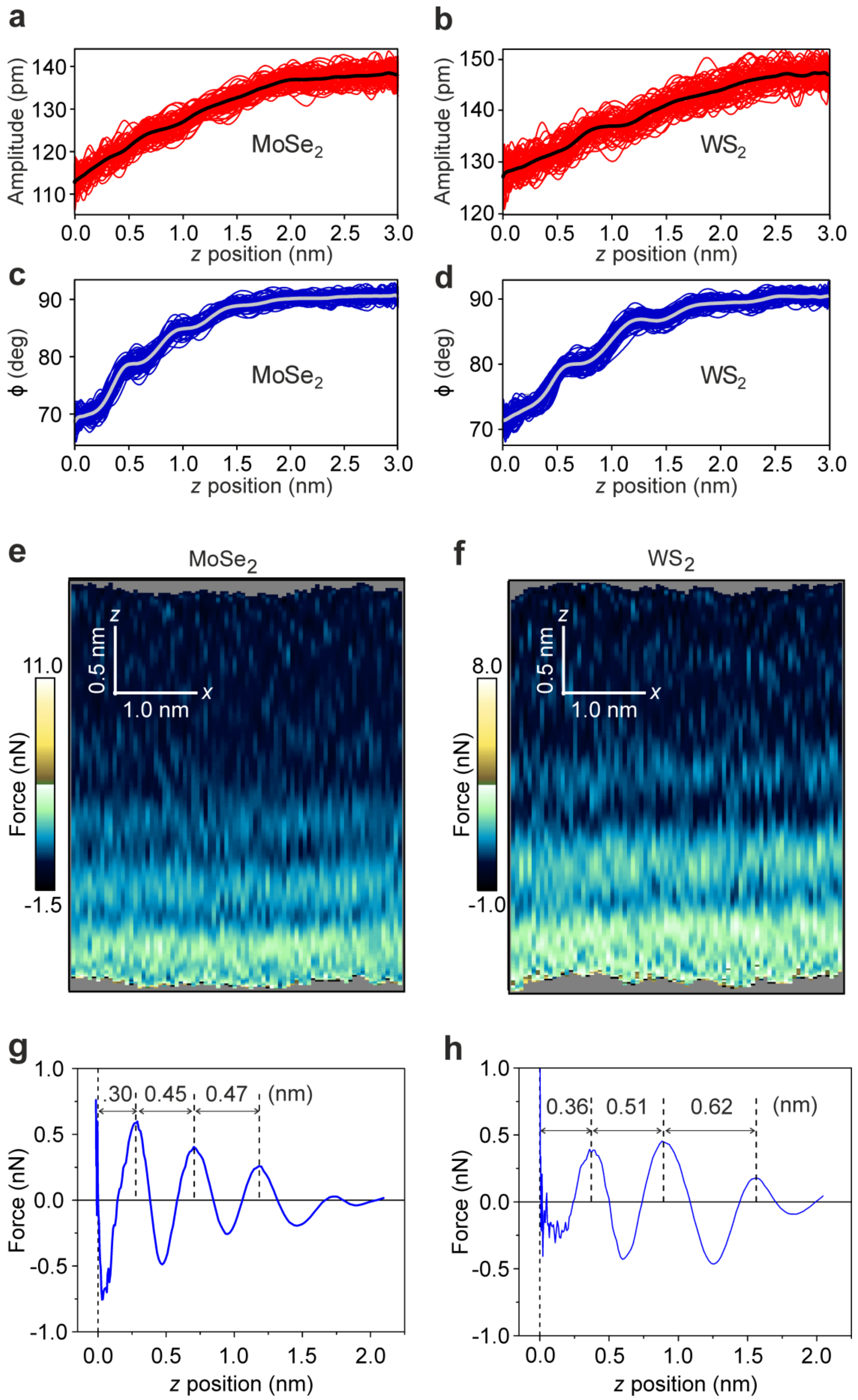

Supplementary Figure 2: 3D-AFM experiments on $MoSe_2$ and WS_2 –water interfaces. (a-d) Amplitude (*A*) and phase shift (φ) *versus* distance curves obtained from the 3D-AFM images. There are 80 curves per panel. The average curve has been highlighted. (e-f) 2D-AFM *xz* force panels calculated from the curves shown in (a-d). (g-h) Average force-distance curve.

Supplementary note 4

Supplementary Figure 3 shows the water contact angle for different materials. We have used a home-built setup consisting of a LED backlight and a high-resolution CCD camera to measure the water contact angle Θ. We have carefully placed 10 µl of ultrapure water on the surface by using a micropipette. The sessile drop contact angle has been measured by fitting a straight line to the gas-liquid contact line. Measurements with asymmetric contact angles (due to visible pinning of the three phase contact line) were disregarded.

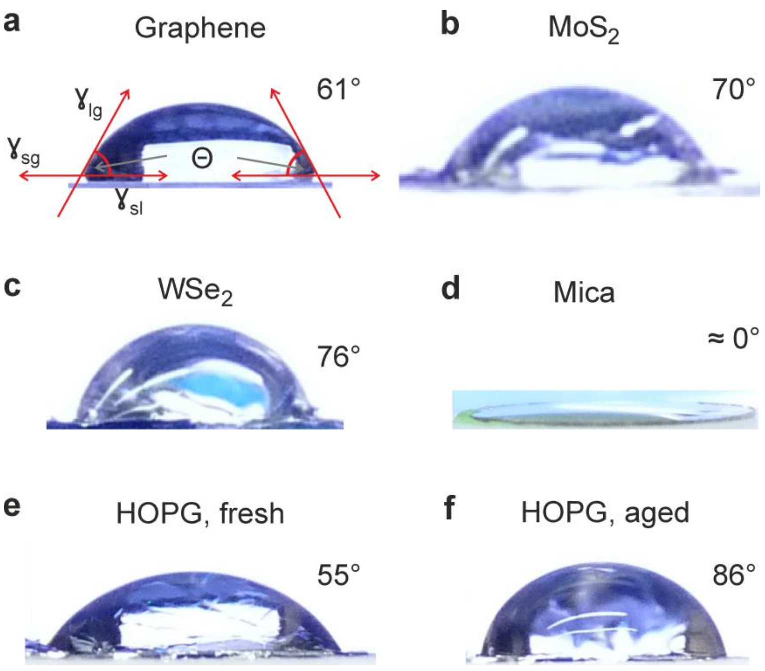

Supplementary Figure 3. Water contact angle measurements. (a) Epitaxial graphene. (b) Bulk MoS_2. (c) Bulk WSe_2. (d) Mica. (e) HOPG (fresh). (f) HOPG (aged, exposed to air for 20 min).

Supplementary note 5

Amplitude and phase shift distance curves are used as inputs to determine the force-distance curves shown in Figures 2 and 3 (main text).

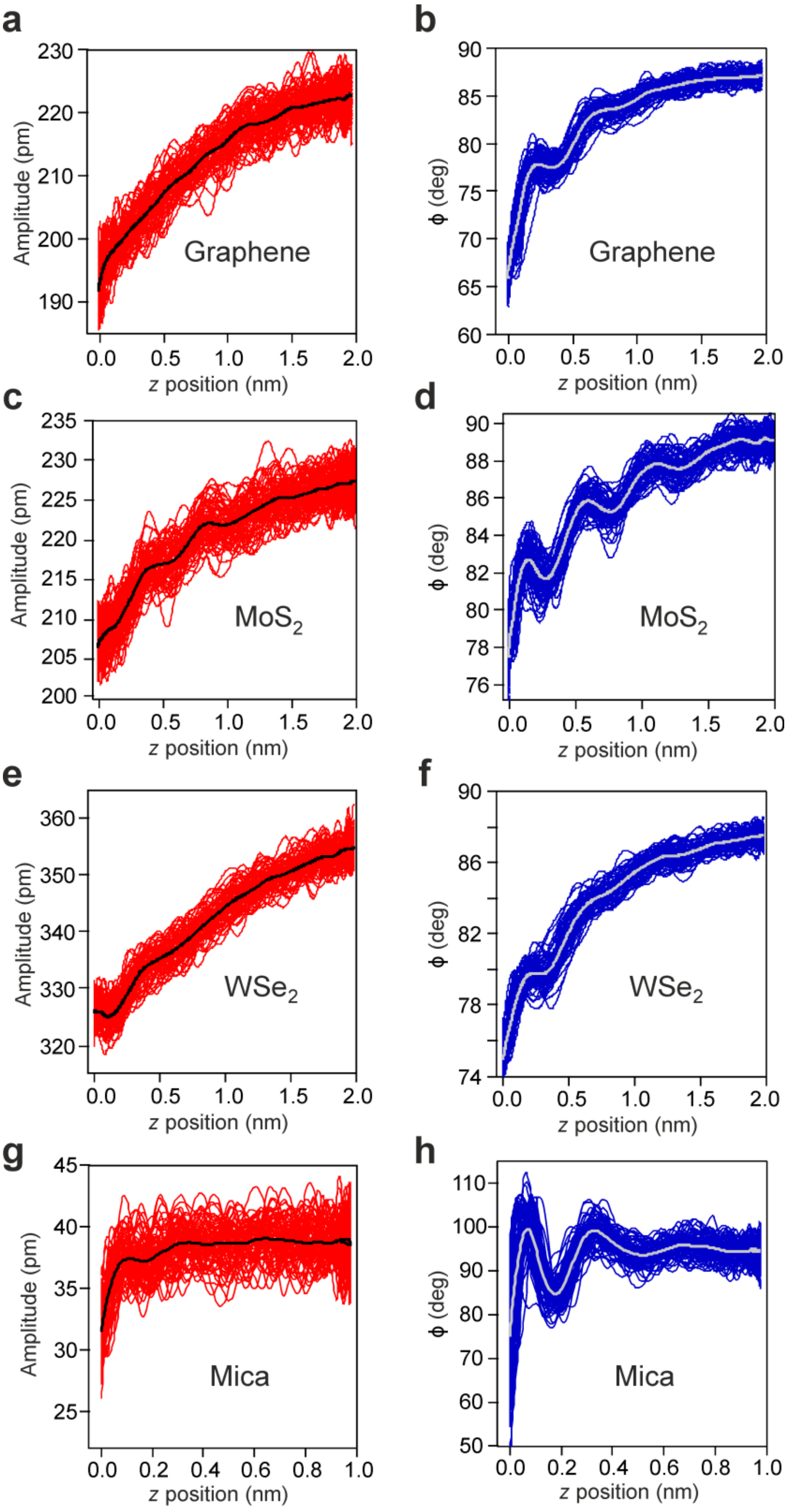

Supplementary Figure 4. Amplitude (*A*) and phase shift (φ) *versus* distance curves obtained in a 3D-AFM experiment. (a) Amplitude- distance curve for graphene. (b) Phase shift-distance curve for graphene. (c) Amplitude- distance curve for MoS_2. (d) Phase shift-distance curve for MoS_2. (e) Amplitude- distance curve for WSe_2. (f) Phase shift-distance curve for WSe_2. (g) Amplitude- distance curve for mica. (h) Phase shift-distance curve for mica. There are 80 curves per panel. The average curve has been highlighted.

Supplementary note 6

Force-distance curves (force curves) are obtained from the observables (amplitude and phase shift) as a function of the tip-sample separation. We have implemented Hölscher's method[1] by using a custom written code (Igor Pro, Wavemetrics, USA). The procedure is illustrated in Supplementary Figure 5. First, we average the raw data over 80 individual curves (Supplementary Figure 4a-b and Supplementary Figure 5a-b). Then, we compute the corresponding force as described in Supplementary Ref 1. Before calculating the derivative, we apply a slight binomial smoothing to avoid spikes due to noise in the raw data. The obtained force curve is shown in Supplementary Figure 5c (in black). For experiments performed in ultrapure water, the force curve consists of a long-range repulsive background with superimposed oscillations. To determine the background's contribution to the force curve, we fit a simple exponential function to the reconstructed force curve

$$F_{bg} = C_1+C_2 \exp(C_3 z) \quad (1)$$

with C_1, C_2, and C_3 as fit parameters. The fitting is shown in Supplementary Figure 5c (in red). Then, the background component of the force is subtracted from the reconstructed force. The final curve is considered to represent the force acting exclusively on the tip apex (Supplementary Figure 5d).

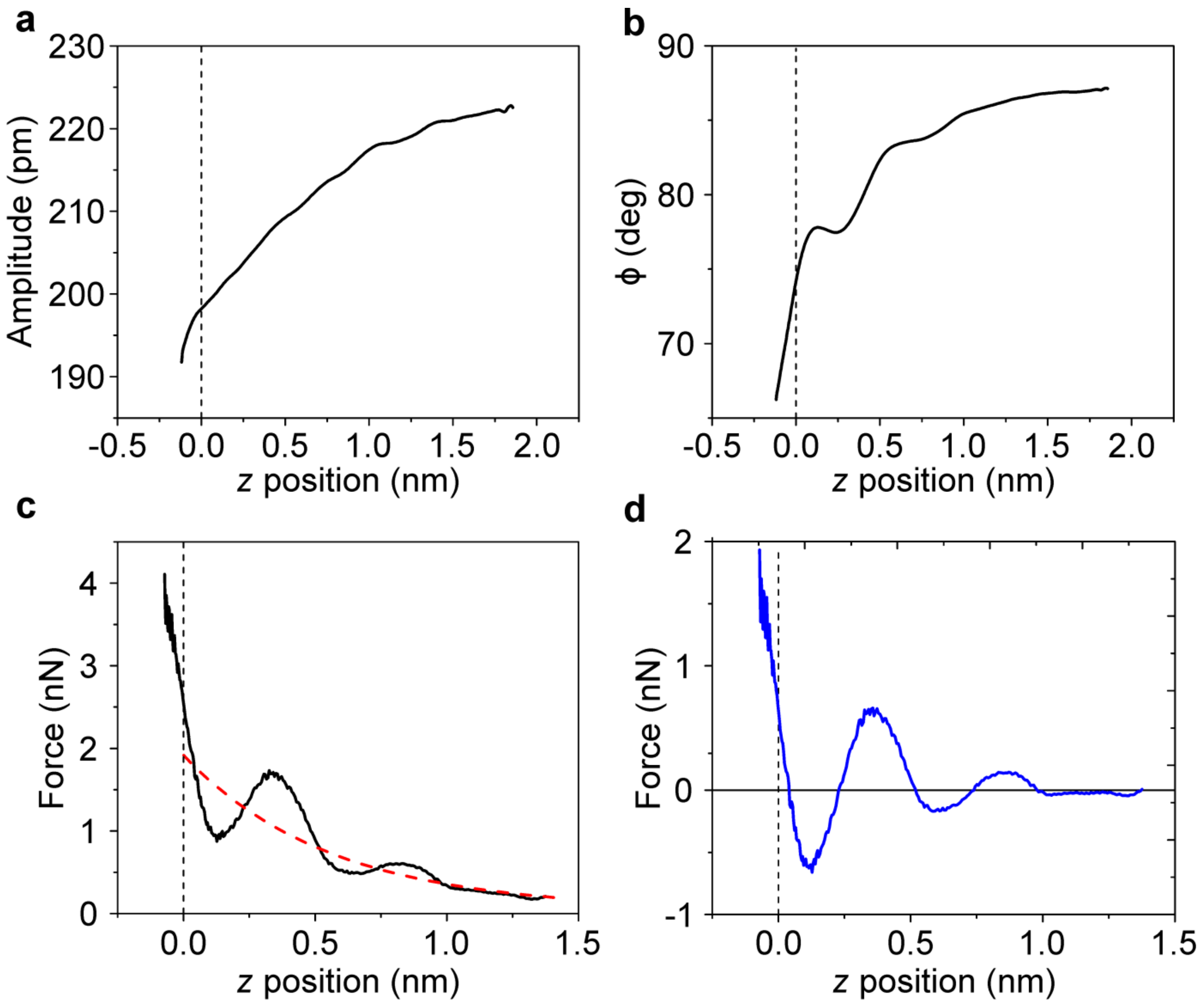

Supplementary Figure 5. Reconstruction of the force curve on an epitaxial graphene surface immersed in water. (a) Amplitude-distance curve. (b) Phase shift-distance curve. (c) Force-distance curve after processing the data from panels a and b. (d) Final force-distance curve after removing the background. The parameters used to reconstruct the force are k_2 = 1126 N m^{-1}, Q_2 = 20.4, f_{r2} = 961 kHz, A_0 = 223 pm.

We remark that the *z* range of the force curve is slightly reduced with respect to the amplitude and phase shift distance curves. This is because the force reconstruction process requires an integration over the oscillation cycle (peak-to-peak amplitude i.e., $2A_0$). The 3D-AFM images have a *z*-depth of $\geq$2 nm. This depth was chosen because we did not observe special features in the liquid structure above $\geq$ 1.5 nm (see Supplementary Figure 10).

Supplementary note 7

In addition to the 2D materials described in the main text, we have also investigated the solid-liquid interface of highly ordered pyrolytic graphite (HOPG) surfaces immersed in ultrapure water. Supplementary Figure 6a shows a 2D panel extracted from a 3D-AFM image. A solvation layer structure similar to the ones obtained on 2D materials is visible. The force curve is shown in Supplementary Figure 6b. The force curve exhibits two peaks with an average distance of 0.5 nm. This is in agreement with previous results obtained on similar HOPG-water interfaces.[2,3]

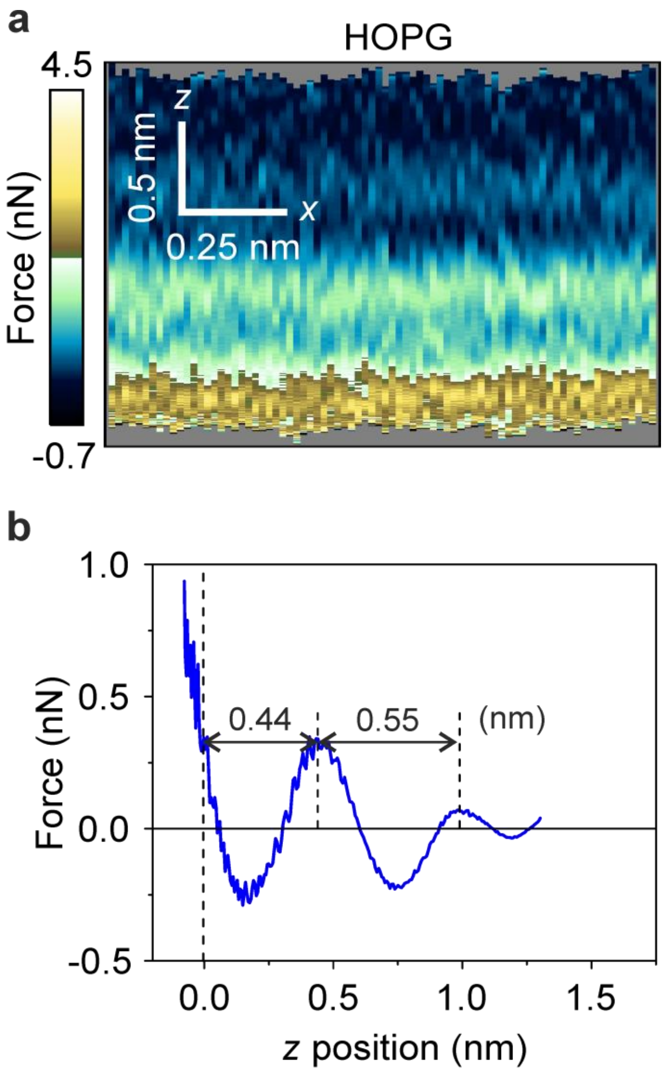

Supplementary Figure 6. HOPG-water interface. (a) 2D *xz* force map. (b) Force-distance curve obtained from (a). The distances between the HOPG surface and the first layer, and between the two adjacent hydrophobic layers are shown.

Supplementary note 8

While imaging some of the surfaces in water we have occasionally observed some periodic structures (Supplementary Figure 7 and 8). Supplementary Figure 7 e-f shows the stripes obtained on SiC/graphene and Supplementary Figure 8 a-b the pattern observed on WSe_2. These periodic structures are independent on the scan size, scan rate and scan angle and, hence, they represent real surface features. AFM phase images[4] (Supplementary Figure 7f) provide an enhanced contrast of the periodic patterns with respect to topography images (Supplementary Figure 7e). A cross-section along the white dashed line drawn in Supplementary Figure 7f is shown in Supplementary Figure 7g. The oscillation shows a periodicity of 4.9 nm. Similar structures have been imaged in AFM measurements on SiC/graphene in ambient conditions[5] as well as on HOPG in water.[2,6-7] They have been attributed to either the adsorption of gasses[2,5-6] or the catalysis of methanol.[7]

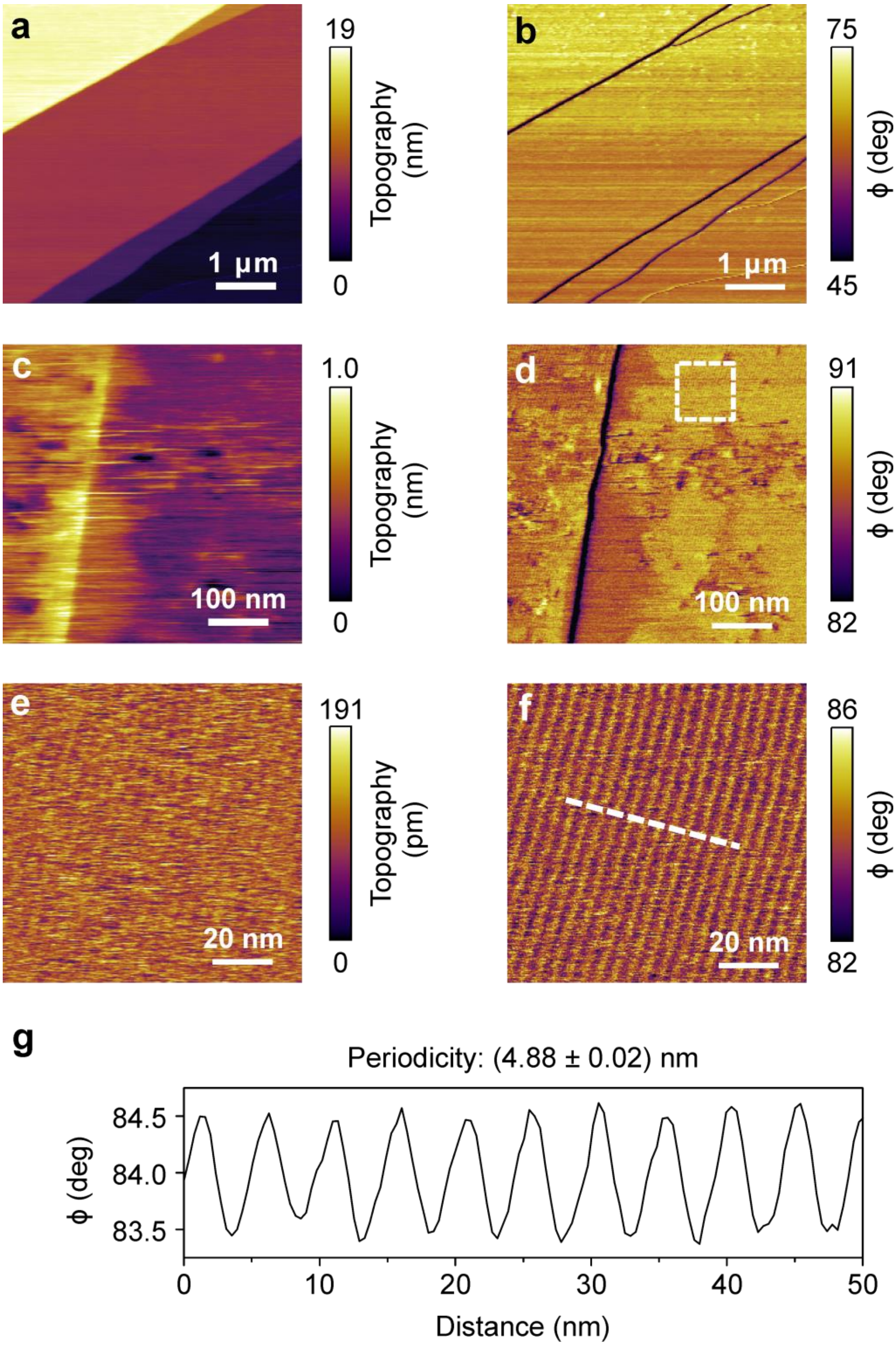

Supplementary Figure 7. AM-AFM images taken in ultrapure water on epitaxial graphene grown on SiC. (a) Topography of a graphene layer with large and atomically flat terraces. (b) Corresponding phase shift image. The surface steps originate from the underlying SiC substrate. (c) Topography image of a different region of the graphene sample. (d) Phase shift image of the region shown in (c). (e) High resolution topographic image of the region marked in (d). (f) High resolution phase shift image of the region marked in (d). (g) The cross section along the white dashed line drawn in (f) gives a period of 4.9 nm. Free amplitude A_0 = 240 pm and amplitude set-point A_{sp} = 0.90 A_0.

Supplementary note 9

Supplementary Figure 8 shows some AM-AFM images of a few-layer WSe_2 flake immersed water. The height image in Supplementary Figure 8a depicts two terraces separated by a monolayer step. The stripe structure of both terraces is visible in the phase shift image (b). The stripes' orientation changes by 90º from the upper to the lower terrace. The periodicity is of 9.3 nm.

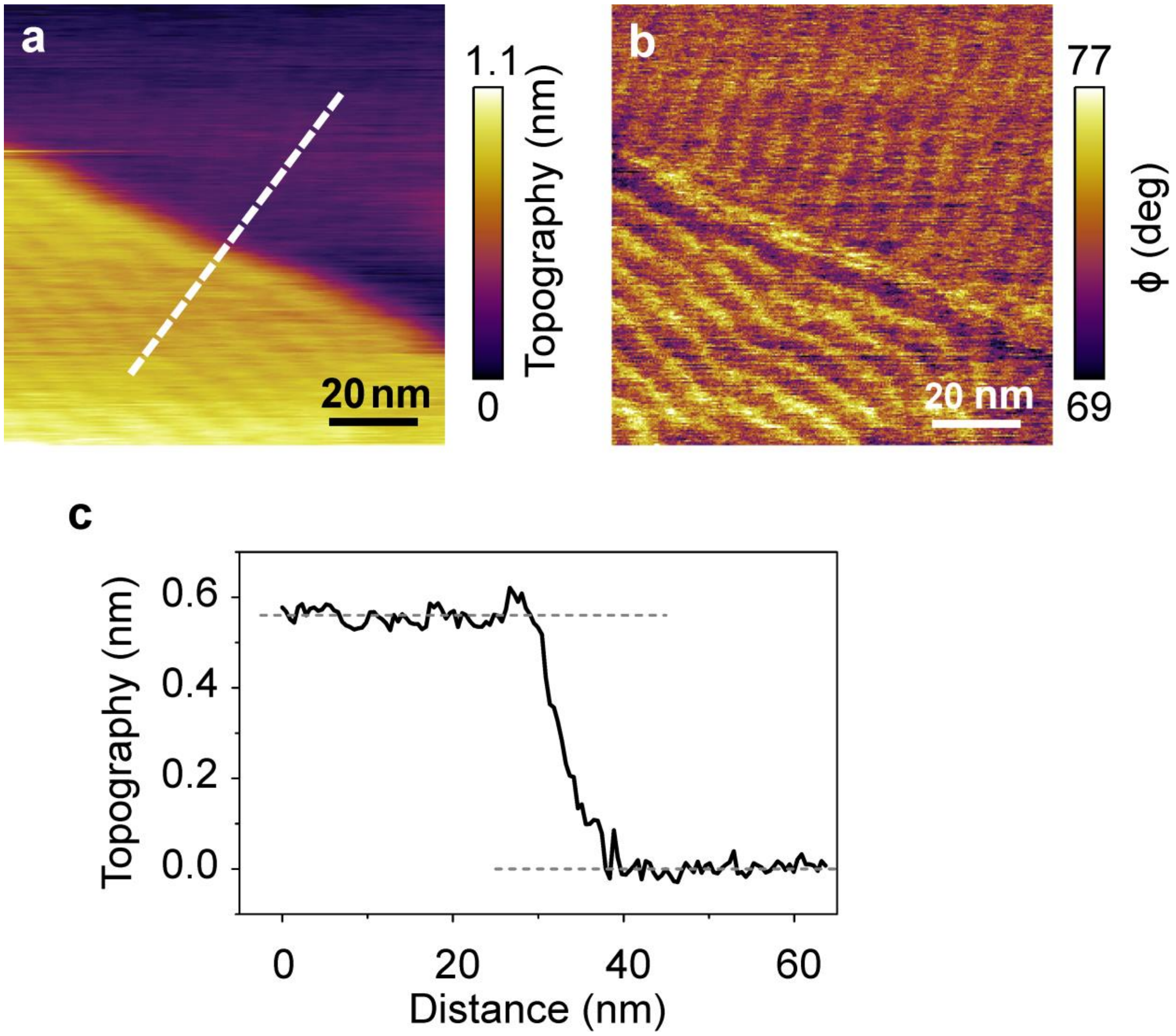

Supplementary Figure 8. AM-AFM images of a few-layer WSe_2 sample immersed in water. (a) Topography. (b) Phase shift. The periodicity is of 9.3 nm. (c) Height cross-section taken along the white dashed line drawn in (a). The AM-AFM was operated with a free amplitude A_0= 240 pm, with an amplitude set-point A_{sp} = 0.90 A_0.

Supplementary note 10

We have performed some 3D-AFM experiments on a few-layer MoS_2 surface immersed in water mixed with *n*-octane (≥99%, Sigma-Aldrich) at a concentration of $c_{n\text{-octane}}$ = 0.7 mL : 1 L.

Supplementary Figure 9 shows a 2D-AFM *xz* force map. The above concentration corresponds to the solubility limit of octane in water. This liquid fits the definition of highly contaminated water. The 3D-AFM data indicates that the number of hydrophobic layers increases when a few-layer MoS_2 surface is immersed in water that has been mixed with a liquid hydrocarbon (*n*-octane). This result strengthens the interpretation of the 3D-AFM data provided in the main text: in the vicinity of a hydrophobic surface, water is expelled from the interface and replaced by hydrophobic layers.

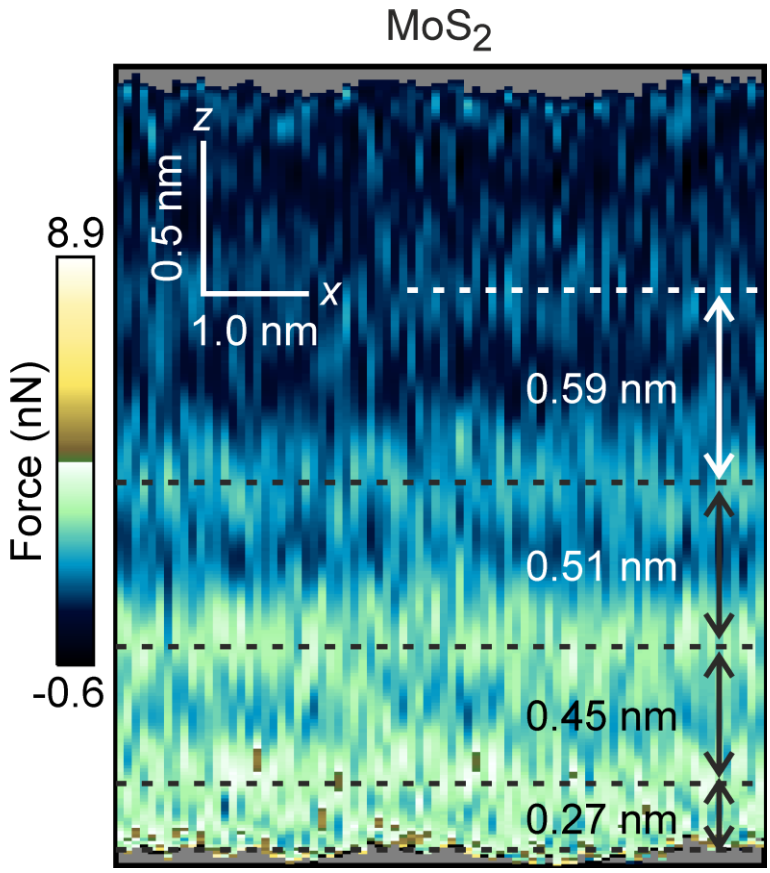

Supplementary Figure 9. A 2D-AFM *xz* force map of the MoS_2-octane:water interface extracted from a 3D AFM measurement. The image shows the characteristic alternation of dark and light stripes observed in purified water. We observe up to 4 hydrophobic layers. The distances between the first two adjacent layers coincide with those obtained with purified water (see Table 1 in the main text).

Supplementary note 11

2D-AFM *xz* force map of a graphene-water interface. The force-distance curve is extracted from the 2D *xz* force panel.

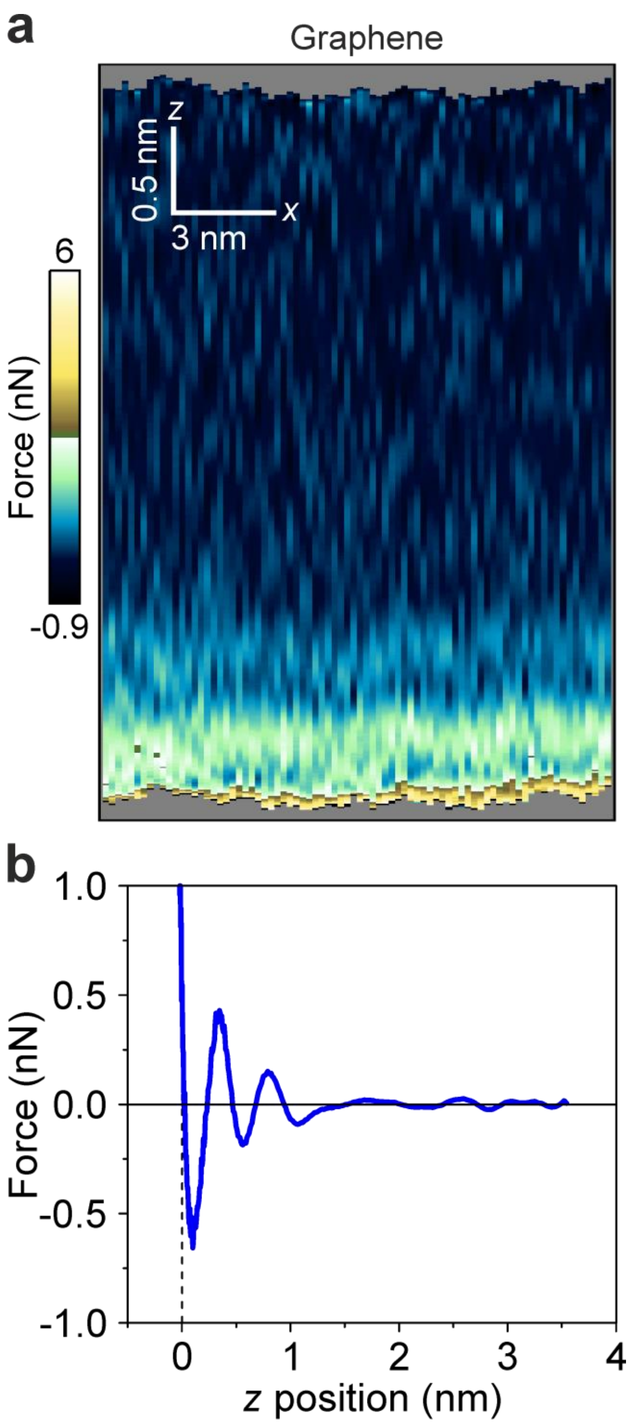

Supplementary Figure 10. Graphene-water interface. (a) 2D *xz* force map. (b) Force-distance curve obtained from (a). The force curve has a steep repulsive section for distances near zero.

An oscillatory behavior is observed between 0.1 and 1.5 nm. No relevant features are observed for tip-surface distances above 1.5 nm.

Supplementary References:

1. Hölscher H. Quantitative measurement of tip-sample interactions in amplitude modulation atomic force microscopy. *Appl. Phys. Lett.* **89**, 123109 (2006).

2. Schlesinger I., Sivan U. Three-Dimensional Characterization of Layers of Condensed Gas Molecules Forming Universally on Hydrophobic Surfaces. *J. Am. Chem. Soc.* **140**, 10473-10481 (2018).

3. Schlesinger I., Sivan U. New Information on the Hydrophobic Interaction Revealed by Frequency Modulation AFM. *Langmuir* **33**, 2485-2496 (2017).

4. Garcia R. *Amplitude modulation atomic force microscopy*. John Wiley & Sons (2011).

5. Wastl D. S., Weymouth A. J., Giessibl F. J. Atomically Resolved Graphitic Surfaces in Air by Atomic Force Microscopy. *ACS Nano* **8**, 5233-5239 (2014).

6. Lu Y.-H., Yang C.-W., Hwang I.-S. Molecular Layer of Gaslike Domains at a Hydrophobic–Water Interface Observed by Frequency-Modulation Atomic Force Microscopy. *Langmuir* **28**, 12691-12695 (2012).

7. Foster W., Aguilar J. A., Kusumaatmaja H., Voïtchovsky K. In Situ Molecular-Level Observation of Methanol Catalysis at the Water-Graphite Interface. *ACS Appl. Mater. Interfaces* **10**, 34265-34271 (2018).
